# Predicting efficacy of drug-carrier nanoparticle designs for cancer treatment: a machine learning-based solution

**DOI:** 10.1038/s41598-023-27729-7

**Published:** 2023-01-11

**Authors:** Md Raisul Kibria, Refo Ilmiya Akbar, Poonam Nidadavolu, Oksana Havryliuk, Sébastien Lafond, Sepinoud Azimi

**Affiliations:** Faculty of Science and Engineering, Åbo Akademi University, 20500 Turku, Finland

**Keywords:** Drug development, Computer science, Scientific data, Drug delivery

## Abstract

Molecular Dynamic (MD) simulations are very effective in the discovery of nanomedicines for treating cancer, but these are computationally expensive and time-consuming. Existing studies integrating machine learning (ML) into MD simulation to enhance the process and enable efficient analysis cannot provide direct insights without the complete simulation. In this study, we present an ML-based approach for predicting the solvent accessible surface area (SASA) of a nanoparticle (NP), denoting its efficacy, from a fraction of the MD simulations data. The proposed framework uses a time series model for simulating the MD, resulting in an intermediate state, and a second model to calculate the SASA in that state. Empirically, the solution can predict the SASA value 260 timesteps ahead 7.5 times faster with a very low average error of 1956.93. We also introduce the use of an explainability technique to validate the predictions. This work can reduce the computational expense of both processing and data size greatly while providing reliable solutions for the nanomedicine design process.

## Introduction

Cancer is a complicated disease caused by abnormal cell growth due to genetic reasons. The severity and societal impact of the disease, along with the fact that effective therapeutics do not exist for many types of cancer, have resulted in cancer therapy being a key area of research for decades. Traditionally, the treatment of cancer has been based on chemotherapy, combination therapy, and radiation therapy, which are effective in some cases, but the toxicity introduced to other normal cells limits the use of these treatments. In contrast, nanotherapeutics provide a more targeted and less invasive alternative. This use of controlled drug delivery has several advantages, including lower dose requirements, greater control over toxicity, and bioavailability of doses^1–3^. The active targeting of tissues is performed using special homing devices, called ligands, with functionalized drug molecules encapsulated within the particle. Apart from this, a large number of other components, such as the size, chemical structure, and delivery method, are involved in the design process of these nanodrug carriers^4^.

A typical nanoparticle (NP) consists of two or three basic layers: the surface, the shell, and the core. Each layer can vary in physicochemical properties such as the shape, size, porosity, hydrophobic properties, or element combinations^5^. As cell-binding moieties, several agents, such as carbohydrates, vitamins, peptides, and proteins, have been shown to work well. Consequently, the process of designing an NP boils down to a rich set of chemical problems with a large number of parameters to explore. Moreover, the particle efficacy is intricately connected to the chosen design specifications^6–9^. This therapeutic efficacy is characterized by the delivery of the drug molecules to their target destinations, as after exposure, they may quickly dissolve before reaching the destination^10^. Often, different statistics derived from configurations such as the solvent accessible surface area (SASA) provide a good understanding of the efficacy and bioavailability of drugs in a certain state^11^. The SASA is designated as the region of the molecule surface exposed enough to be able to interact with solvent molecules. Hence, the design of an NP must constitute the physicochemical properties that lead to a higher SASA value through their biological interactions^12^. However, exploring the vast parameter space and identifying designs with target characteristics is a large limitation both in terms of time and cost.

A more efficient and reliable way to find a good design is to use molecular dynamics (MD) simulations. Through MD simulations, hundreds of atoms with biological relevance can be included in a design, such as entire proteins in a solution with explicit solvent representations, membrane-embedded proteins, or large macromolecular complexes such as nucleosomes or ribosomes. MD simulations allow in silico modelling of the cellular uptake and intracellular trafficking of NPs. In addition, these models provide data for monitoring NP interactions as they enter and exit a cell, which are difficult to calculate otherwise^13^. Internally, simulations make use of the forces acting on every atom. This can be obtained by deriving complex equations and deducing the potential energy from the molecular structure. However, the complex equations of MD simulations create two principal challenges^14^. The first challenge is to derive the potential energy for the system. There is a need for further refinement because the simulations are poorly suited to certain systems. The second challenge is the high computational demand of the simulations, which prohibits routine simulations with lengths greater than a microsecond. This leads to an inadequate sampling of conformational states^15^.

One way of accelerating MD simulations to take advantage of advanced hardware technologies such as graphics processing units (GPUs)^16–18^. A GPU provides higher performance than a single CPU core in terms of increased speed and overall processor utilization. However, GPUs lack the flexibility in their hardware architectures to implement all MD simulation algorithms. Extensive rework and optimization must be applied depending on the specific algorithm to enable it to work efficiently on these specialized pieces of hardware.

The limitations of hardware architecture can be resolved using machine learning (ML) during the development of MD simulations and molecular modelling. Wang et al. reviewed the use of ML-based methods to analyse and enhance MD simulations^19^. The first use of ML was to analyse the high-dimensional data produced by MD simulations through the use of artificial neural networks (ANNs). Different forms of ANNs can be used to produce latent vectors in a low-dimensional feature space from trajectory data. This enables an efficient way of evaluating the equilibrium and dynamic properties of systems^20–28^. Another set of studies focuses on the active involvement of ML-based techniques during the simulation process to improve the sampling time and capacity^29–47^. However, for both objectives, model interpretability or model transferability to new systems poses a challenge. Another recent work implemented distance-based ML algorithms to simulate the atomistic interactions of a $Au_{\text {38}}(SCH_{3})_{\text {24}}$ nanocluster. The presented solution involves the use of transformation techniques to convert atomic coordinates into vectors of atomic interactions through descriptors that can be directly used with ML models. A Monte Carlo strategy was used to evaluate the energy landscape learned through the ML models and showed great results. However, the models were trained solely with $Au_{\text {38}}(SCH_{3})_{\text {24}}$ nanoclusters and focused mainly on a faster configuration space probing method. Hence, a study that can predict some target metrics for NP designs, such as the SASA value, without running MD simulations over a longer period and is generalizable to new systems holds much significance.

In this study, we propose a twofold approach. On the one hand, the issue of applicability of models to new NP designs is tackled, and on the other hand, using explainable AI provides a way to interpret the results. The proposed solution consists of three steps: transforming the data, using a hybrid ML network to predict the SASA value at a specified timestep, and using feature importance to explain and validate the results. Experimental atomic coordinate data for different NP designs are derived from MD simulations and are transformed using the many-body tensor representation (MBTR) descriptor, which reduces the data size and complexity, as well as reflecting interatomic interactions between pairs of elements. We present a combined ML system that consists of a time series model used to simulate the MD interactions over a specified period and a second deep neural network (DNN)-based model to calculate the SASA metric from the intermediate state. Feature importance is calculated using SHAP values to reflect the contribution of each element pair’s interactions. In this paper, we show that ML methods can be used to substantially reduce the cost of NP simulations and, consequently, provide an efficient assistive tool for exploring the NP design space. This work is a novel study of predicting the SASA as a representative example; however, the approach can be generalized to a wide range of other properties and different molecules as well. In addition, we introduce a way to provide explanations for the models that increases both the reliability of the model and can give insights into better NP designs.

## Results

**Figure 1:**
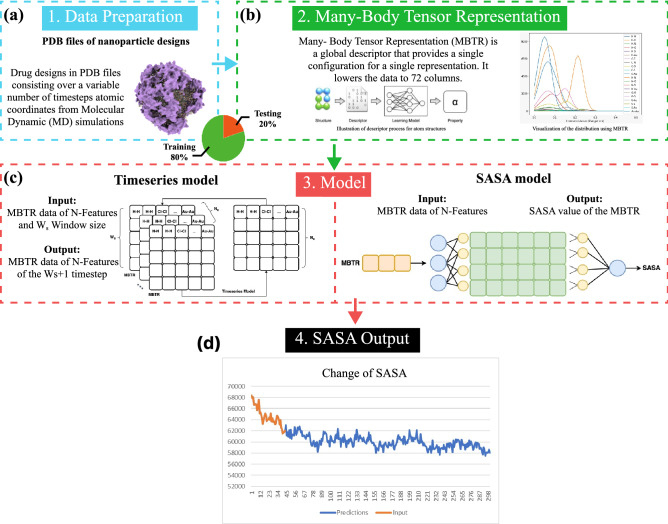
ML-based hybrid solution for the prediction of SASA values (**a**) Atomic coordinates for different NP designs derived from MD simulations in an aqueous environment. These data are in Protein Data Bank (PDB) format with other information such as the respective residues. (**b**) ML usable MBTR representation of the data extracted through a geometric function of pairwise distances between elements. (**c**) Time series model to accomplish the task of simulation and SASA model for the calculation of the target label. (**d**) A predefined batch of data can be used to forecast changes in the SASA. An optimal value for the size of this predefined batch can be set with consideration to the simulation costs for generating them and the error threshold (see Table 1). Although both the input and output of the models are the MBTR vectors except the final output, the graph represents the input-output relationship only.

The data used in this study are snapshots from MD simulations involving NP designs functionalized with 9 different drug types (see Table 3). These snapshots were taken over a range of variable periods at a rate of one snapshot per nanosecond. Specifically, 64 NP designs were recorded over 300 ns, 32 were recorded over 200 ns and 23 were recorded over 120 ns. These snapshots contain the Cartesian coordinates of the atoms in the systems along with other information and represent how the atom movements are dictated by the environment. We first transform these data into vector encoding by extracting design-specific global properties through MBTR descriptors. As a result, the data become manageable and compressed with only ($n_{\text {features}} =$) 72 features representing each state. In order to apply ML models for the prediction of SASA values at future timesteps, the proposed solution combines two different models, each responsible for a part of the overall objective, as illustrated in the proposed workflow in Fig. 1. These are:

1. **Time series model:** This model is used to learn the inherent properties from a fixed window of MBTR vectors that influence atomic interactions during the period. This learned pattern is used to forecast future MBTR vectors and used in a sliding window mechanism until the vector for the specified time is predicted. Hence, this model enables the approximation of the state of an NP at any given point in time in the future.
2. **SASA model:** To calculate the SASA value by exploiting the transitive property between the atomic coordinates and the MBTR vectors, we use a second model. This model predicts the $\vert SASA^{\langle t\rangle }\vert = P(\theta \vert V^{\langle t\rangle }_{\text {MBTR}})$ value for any particular timestep, *t*, where $\theta$ is the learned parameter.

The data are split into training and test sets with a ratio of 80:20, which translates to 107 designs in the training set and 12 in the test set. During the splitting, the order in time for the data of each design is preserved for the models to capture the sequential properties. The range of SASA values for different designs varies greatly; hence, the test set is manually chosen to have representative samples from different ranges in the dataset. Each of the 12 designs in the test set along with the whole training data is depicted in Fig. 3a by taking the minimum and maximum SASA values over the whole period.

### Time series prediction

As discussed in the “Methods” section, we experiment with two approaches for time series prediction. Both approaches process the input data based on a sliding window method, and the window size dictates how long the simulations run before a solution can be used. The first approach is a transformer model using multivariate MBTR vectors as input to predict the next timestep’s MBTR. The transformer model is used because the self-attention mechanism of the model is suitable for effectively approximating the interatomic interactions. The model achieves a mean absolute error (MAE) value of 40.16 on the test set for 3120 test samples for a fixed window size of 40. Here, we use the MAE as the error metric since it provides a linear score for deviation from the original value in a compact scale. The final MAE values are much higher than expected, which can be attributed to the smaller size of the dataset for such a large model. Hence, we use the second approach to minimize the error values with the same amount of data.

As the next method, an ensemble approach is trained using 72 separate XGBoost models^48^, with each model predicting the value for the next timestep of each feature. The outputs from each model are then concatenated to produce the final vector for that timestep. The results of how different values of window size influence the outcome of the ensemble approach are presented in Table 1, and in all cases, the MAE value is comparatively much smaller and suitable for the solution. The best achieved MAE of 1.57 is for the smallest tested window size of 10.

**Figure 2:**
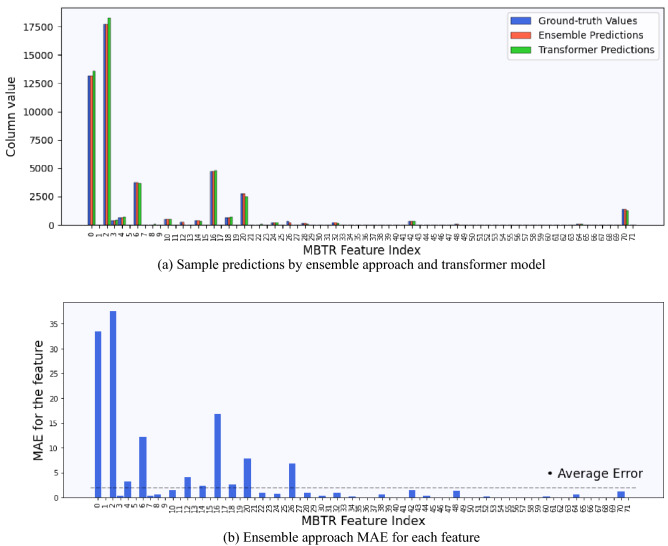
Time series model performance. (**a**) A scalar value predicted by each model from the ensemble approach and multivariate prediction by the transformer model for a sample data pair of the *NCL11* NP design from the test set. The differences between the heights of the bars representing the predictions and the ground-truth values indicate the prediction errors. (**b**) MAE for each XGBoost model from the ensemble approach over the whole test set. The dashed line represents the average error across all features.

Figure 2a shows the bar plot representation of the predictions using the ensemble approach and the transformer model for a randomly sampled test data, respectively. Figure 2b shows a detailed bar plot representation of the MAE for each model from the ensemble approach.

From the predictions, it can be seen that the XGBoost models provide better accuracy than the transformer model. From Fig. 2b, we can see that most of the features in the ensemble approach produce below average MAEs. There are 8 features that have above average MAEs, while only 4 features out of the 72 have an MAE above 10.

With these results, we used the ensemble approach as the time series predictor for the combined solution. Additionally, as this approach uses a classic ML algorithm, it is robust to smaller dataset sizes.

### SASA prediction

To determine the best performing deep neural network model for this task, models with different architectures are evaluated. Keeping the number of layers and activation functions the same, we experimented with different numbers of neurons in the feedforward network. The model with 512 neurons in each hidden layer had an MAE value of 6265.85, whereas the model with 128 neurons had a higher MAE value of 6810.92. In contrast, the model with 256 neurons in each hidden layer had the best performance, with an MAE value of 936.42; hence, it is used as the base model.

**Figure 3:**
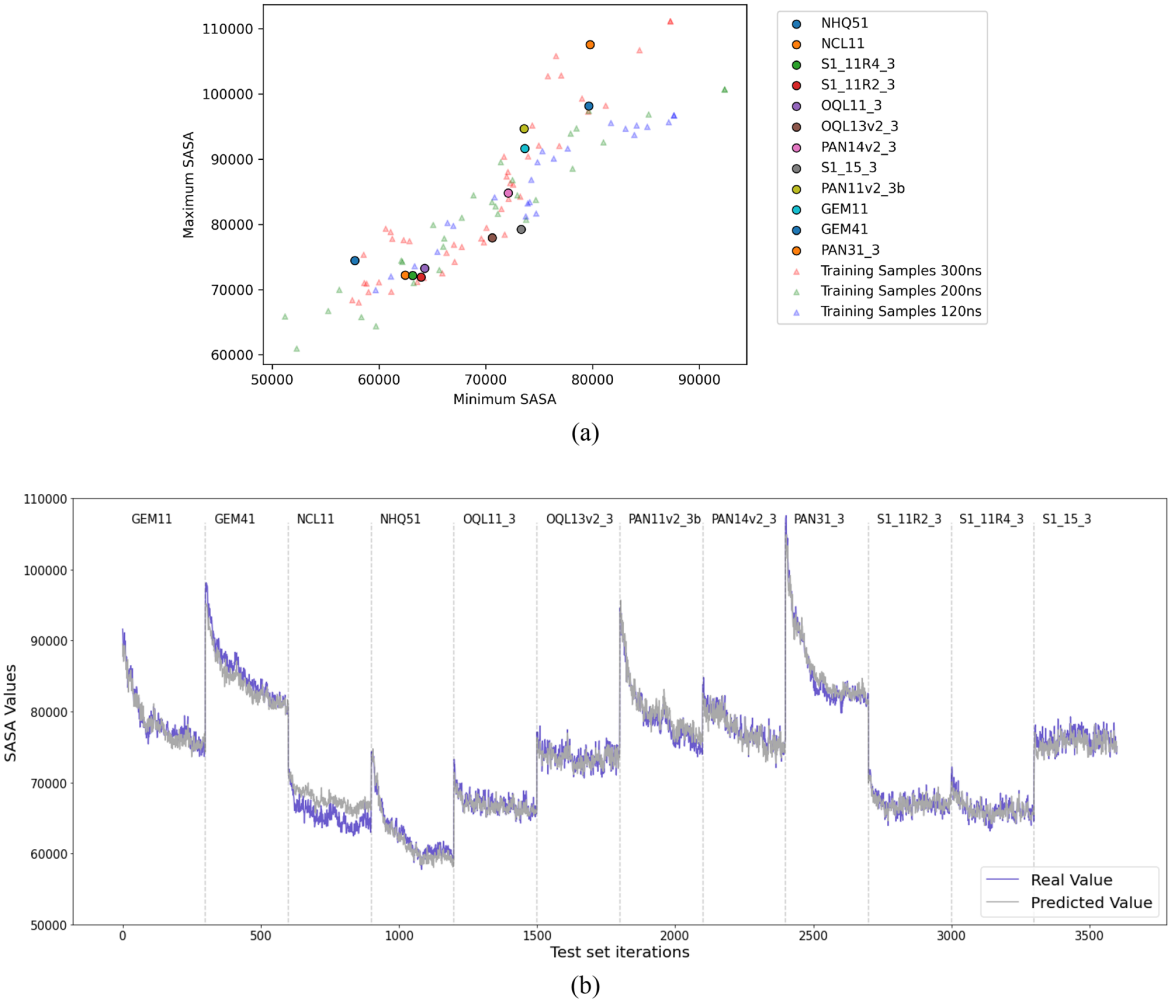
(**a**) The distribution of NP designs in the training and the test set based on the minimum and maximum SASA values. Each dot represents test data for a particular NP design, with the letters of the design name referring to the drug type and the remainder being a unique identifier. The training samples are represented by triangles and grouped by their sizes. (**b**) Visualization comparing the real and predicted SASA values of different NP designs (separated by dashed lines) from the test set over 300 iterations each. The blue line represents the actual SASA value, and the grey line represents the predicted SASA value.

Both the MBTR vectors and the SASA values of the NP designs for each timestep were stacked vertically for the training and testing datasets. Figure 3b illustrates the predicted and expected SASA values that change continuously for 300 iterations of different designs in sequential order.

The model can learn the range of SASA values for each design and how the SASA values decrease over time. The model can generalize well to new or unseen data as well. As seen in Fig. 3b, after encountering a new design every 300 iterations, the model quickly adapts to changes in the SASA.

### Combined inference

As the SASA value takes an uncertain amount of time to reach a stable range, the duration for MD simulations has to be predefined to a maximum value during which all NPs are expected to reach that state. Reflecting the same property, inferences in the proposed solution can be made for a given amount of time, which is achieved by running the time series model $s_{\text {steps}}$ = $t - w_s - 1$ times, where *t* is the target timestep. We start the combined inference with the MBTR vectors of the initial timesteps for a fixed window size and use the proposed workflow to predict the SASA value at the 300th timestep. Different window sizes, $w_s$, are tested, the same as those for the time series model, and the results are evaluated by comparing the actual SASA value at the 300th timestep for the design and the predicted value using Eq. (1). The comparative results are demonstrated in Table 1.

1 $$\begin{aligned} error_{\text {SASA}} = \frac{1}{k} \times \sum _{i=1}^k \vert \hat{y}_i^{\langle \text {t}\rangle } - y_i^{\langle \text {t}\rangle } \vert \end{aligned}$$

where *k* refers to the number of NP designs in the test set, *t* is the final timestep for that design, $y_i$ is the ground-truth and $\hat{y}_i$ is the predicted value for the *i*th design.

**Table 1:** The impact of different window size values, $w_s$.

Window size, $w_{s}$	Training time for ensemble approach (s)	Ensemble approach MAE	$error_{\text {SASA}}$
10	44.60	**1.5692**	2128.62
20	46.46	1.6124	2172.40
40	49.63	1.7294	**1956.93**
60	59.97	1.7781	2399.88
80	63.48	1.7955	2298.94

The lowest values are in bold.

From Table 1, it can be observed that although the MAE for the time series model is smallest in the case of a smaller window size, the best score for the combined inference is achieved with a window size of 40. Hence, we use this value for comparing the outputs for the test set designs with respect to ground-truth values acquired by MD simulations, and the results are presented in Table 2.

**Table 2:** Comparison between the predictions for the test set and the ground-truth values. Both the predictions and the actual values are for the 300th timestep of the test set designs.

Design name	Predicted SASA	Predicted change$^{\text {a}}$ (%)	Actual SASA	Actual change$^{\text {a}}$ (%)
GEM11	78,390.13	86	74,784.69	82
GEM41	83,150.56	86	80,337.07	83
NCL11	62,458.61	86	63,675.28	88
NHQ51	59,342.53	80	60,745.86	82
OQL$11_3$	67,595.29	92	66,567.95	91
OQL13v$2_3$	71,308.36	93	75,432.52	98
PAN11v$2_{\text {3b}}$	77,509.73	82	74,523.94	79
PAN14v$2_3$	75,104.65	90	76,080.15	91
PAN$31_3$	83,197.91	78	82,504.89	77
S1_11R$2_3$	67,374.14	95	68,690.32	97
S1_11R$4_3$	67,374.28	95	64,980.52	91
S1_$15_3$	74,141.72	99	75,070.26	101

${}^{\text {a}}$The change is the ratio between the SASA value at the target timestep ($t = 300$) and the initial SASA value ($t=0$) for the design.

It can be observed from Table 2 that the predictions are very close to the SASA values achieved through running MD simulations for the whole duration. As a result, the potential of the model is large, especially considering the computing and resource expenditures of acquiring the values through MD simulations for a large number of NP designs.

### Explainable AI prospects

To establish the reliability of the results, we use SHapley Additive exPlanations (SHAP)^49^. It is applied to our model to obtain the importance of the atomic interactions that greatly affect the model’s output, i.e., the SASA value. From the results of the proposed approach, we can observe a strong correlation between the MBTR descriptors and the corresponding SASA values. This indicates that the interatomic distances can impact how the NP evolves.

Since the same structure from different residues may have different effects on solubility, the whole drug-carrier system is not suitable for determining feature importance . For example, *Panobinostat*-based and *Quinolinol*-based NPs have opposing properties: Panobinostat is a hydrophilic (attracted by water) drug, whereas Quinolinol is hydrophobic (repels water), which have different impacts on the resulting SASA value^7^. As the drugs have the same groups consisting of the same elements, using the relation between interatomic distances created by the MBTR and their SASA values is insufficient for explanations. For this reason, we generated MBTRs and built a separate model for each residue. In our approach, we focus on explanations for each residue to provide the pair of elements within them, which can result in a higher SASA value, as opposed to elements that are less significant.

**Figure 4:**
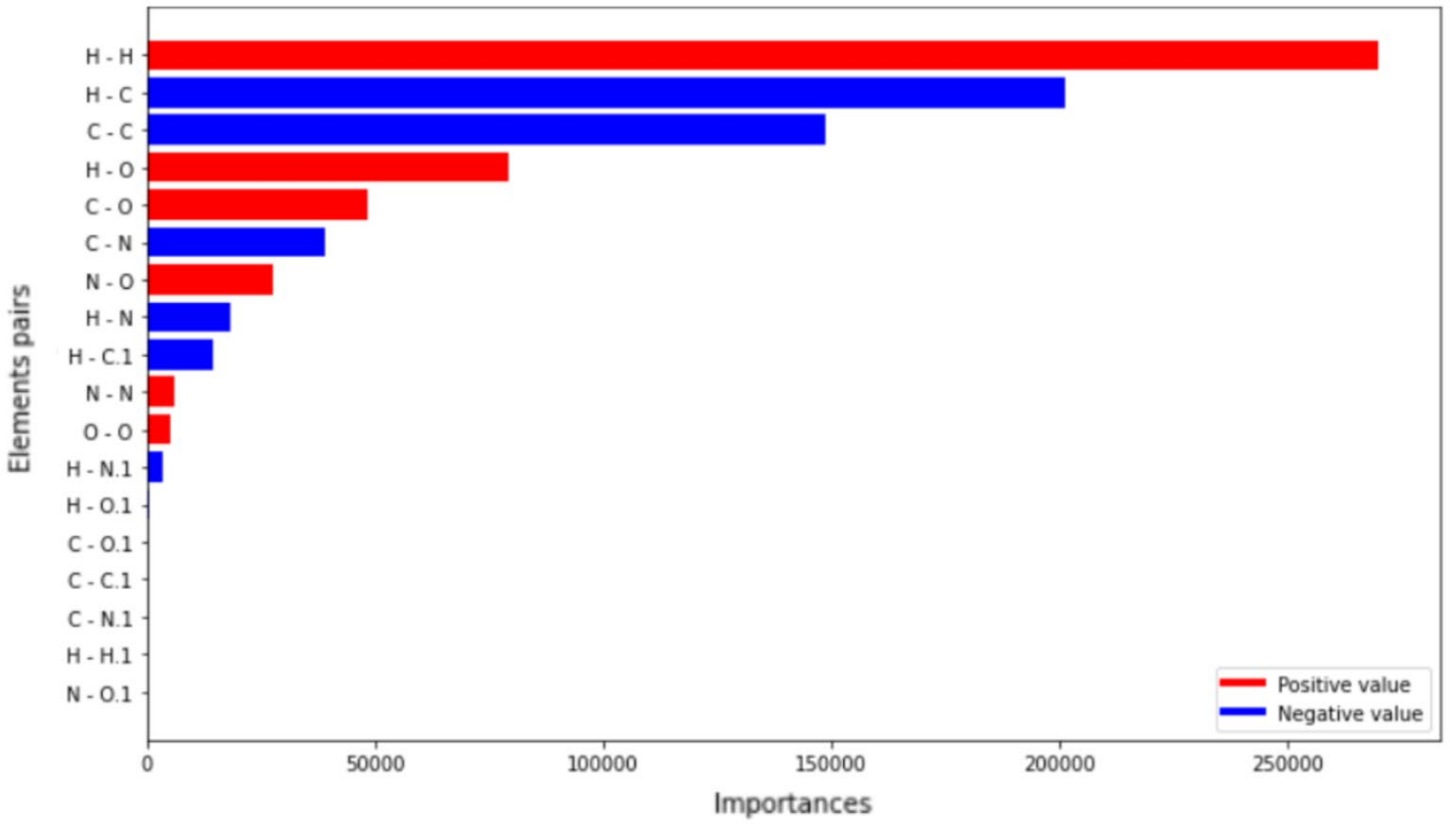
Feature importance graph for the *Panobinostat* residue from 14 NP designs that carry the drug. The element pairs are put in decreasing order by importance starting with the most influential one. The red and blue colours indicate positive or negative impacts on the resulting SASA value, respectively.

For example, for the drug residue from *Panobinostat*-based NPs (Fig. 4), it can be observed that pairs of hydrogen atoms and carbon atoms are very important in terms of how steady the molecules on the surface are. The graph shows both positively and negatively affecting element-pairs. Positive interactions can lead to an increase in the SASA value, whereas negative interactions can lead to a decrease. The phenomenon of hydrogen atom pairs having such a large impact may be because the more spread the hydrogen atoms are, the greater they can create hydrogen bonds with the solvent molecules. In contrast, as the carbons exist mainly in long chains, a relatively higher distance may indicate folding, which reduces solubility.

## Discussion

Due to the wide range of biochemical and physicochemical properties of NPs and the expensive in vivo testing process, computational solutions (often MD simulations) are more feasible and precise for the study of NPs in anti-cancer treatment^50^. This work has been developed within an application scenario defined in the H2020 project EVO-NANO. The overall project scenario was to perform in silico NP design evaluations (MD simulations) before the synthesis of selected NPs, the evaluation of the designs via in vitro experiments using vascular microchips, and finally in vivo experiments using mouse cancer xenografts in which biodistribution, efficacy, and toxicity of the designs can be validated. Although computational methods provide a faster way to transition from the laboratories to the clinical field, they have the bottleneck of high computational resource and time requirements that limit the experimental possibilities. The work presented in this paper focuses on the in silico step and proposes an approach to accelerate the evaluation of NP designs by predicting the stable state without the need to execute complete MD simulations.

The most significant contribution of this work is that it addresses the limitations of MD simulations and provides a scalable solution. It presents the opportunity of eliminating NP designs that do not possess the expected properties from the large pool of designs. As a result, a selective number of drug-carrier systems can be chosen with the largest efficacy values for further assessment. It takes several days to complete an NP simulation over 300 ns using high-performance computing resources, while the approach discussed in this research takes less than ten minutes to complete, starting from the input batch. Hence, if $w_s = 40$ is used, the time gain is approximately 7.5 times (300ns simulation time / 40ns simulation time) for a simulation period of 300ns. The cost of the computation can also be solved since the trained model can be used to predict the stable state of the NP design within a very short amount of time, while the simulation steps are adjustable. Real case studies on the use of automated learning method-based prescreening processes have already shown to be feasible and accurate^51^, whereas the target variable, SASA, has been observed to be effective for comparative analysis between different configurations of NPs^7^. In addition, this approach can be adapted to other related applications where certain properties must be monitored, such as hydrophobic/hydrophilic properties^52^.

In drug discovery, explaining decisions made through ML models is crucial, especially based on the impact. Some of the most important properties of such explanations are transparency—to understand the rationale behind the predictions, justification—the reasoning behind the acceptance of the outcomes, and informativeness^53^. An explainable outcome not only establishes the credibility of the results through validation of what is expected but can also be used in the reverse way to find any association between the molecular structure and the physicochemical properties. We use local explainability techniques and demonstrate feature importance for a subset of the problem to achieve transparency. The effect on the target property for relative interatomic distances may not be directly applicable in the design process, but it can be used to establish new insights into the relationship between molecular structure and the target property. Moreover, information can be expanded by breaking down the problem into finer pieces and observing the model’s behaviour from every perspective.

A limitation of this work is the limited availability of the training data. Having varied data with different SASA ranges can enhance the model performance. Currently, the model has been trained with 107 different designs, and having exposure to new designs can help the model generalize more. Another limitation is the use of the MBTR descriptor, which encodes the whole NP structure into a simpler form at the cost of information loss. In the future, instead of working with a single descriptor, implementing a combination of different descriptors can help summarize the complex structure in a concise form without losing any properties of the NPs. Additionally, we have explored explainability in this work in a limited scope and demonstrated that the potential of such techniques in this area is very large. However, the relative distances between atoms are not configurable; hence, they cannot be translated to design decisions. As a future recommendation, explanations can be expanded in a way that every structure from an NP design can be thoroughly assessed and can directly influence the design decisions. This can be achieved by extracting a hierarchy of properties, for instance, the ratio of drug to background molecules, the number of residues, and the size of the NP and the core, and evaluating the target characteristics against those.

## Methods

In this section, we discuss the data used for this study, the transformation technique, and the proposed models in detail. This study did not require ethical approval.

### Data description

**Figure 5:**
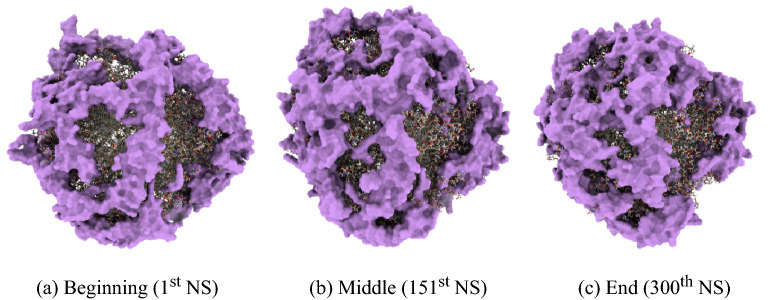
Simulation figure for a design containing the drug Panobinostat, generated using ChimeraX^55^. The purple portion depicts the drug molecules around the surface.

The data we use in the project are derived from MD simulations which are generated using AMBER19 software^54^. In these simulations, the initial energy of the systems was minimized, and then the temperature was increased to 300 K. The MD simulations were run for one NP design at a time and stored in PDB format, which is a standard for files containing atomic coordinates. A PDB file contains information about elements used in the system, atomic coordinates in *(x, y, z)* format, and residue names. A simulation was run for some predefined time, which in this case was 300, 200, and 120 ns. When the MD simulations for a particular NP design were being run, the PDB files were extracted at 1 ns intervals. An example of simulation states in the beginning, middle, and end of the simulation is shown for a Panobinostat drug-based NP design in Fig. 5.

A gold (*Au*) core is used in each of the systems, as it provides a low toxicity level and inertness and is easy to produce. The systems are designed with one of 9 different drug types, which can be classified either as hydrophobic or hydrophilic with respect to each other. These NPs are functionalized through ligands such as polyethylene glycol, dimethylamino, and amino groups. The systems contain 6 or 7 unique elements, including *Au*, *S*, *H*, *C*, *O*, and *N*, and can additionally contain *F* or *Cl*. Apart from the drug molecules, other residues are used in combinations of 5–7 different types per NP. The drug-forming residues are described in Table 3.

**Table 3:** Description of the drug-forming residues.

Residue	Chemical identity	Hydrophobicity$^{\text {a}}$	Elements
CY5	CY5	Hydrophilic	*O, C, H, N*
DOX	Doxorubicine	Hydrophilic	*O, C, H, N*
GEM	Gemcitabine	Hydrophilic	*O, C, H, N, F*
NCL	Niclosamide	Hydrophobic	*O, C, H, Cl, N*
NHQ	Quinolinol	Hydrophobic	*O, C, H, N*
PAN	Panobinostat	Hydrophilic	*O, C, H, N*
WYC	Wyc-215	Hydrophilic	*N, H, C, O, S*
ZIL	Zileuton	Hydrophobic	*O, C, N, H, S*
ZOR	Zorac	Hydrophobic	*N, H, C, O, S*

$^{\text {a}}$This property is not absolute; hence, it is determined based on whether the molecules are more hydrophobic or hydrophilic.

A comprehensive discussion on how the NPs were designed for this experiment along with how the simulations were conducted is presented in the study by Kovacevic et al.^50^ For calculating the ground-truth total SASA values of the corresponding timesteps for each of the NP states represented by the PDB files, Visual Molecular Dynamics (VMD) program was used^56^.

### Transforming the data using descriptors

To make the data suitable for application to an ML algorithm while keeping the representations computationally inexpensive and robust to rotations, permutations, and translations, we use MBTR descriptors. An MBTR is a global descriptor that provides a unique representation for any single configuration^57^. Each system is divided into contributions from different element pairs and described using relative structural attributes. In this work, to extract a single value conforming to a particular configuration of *k* atoms, we use an inverse distance-based geometric function, $g_2$, as in Eq. (2). The structure is then represented by constructing a distribution, $P_2$, of the scalar values using kernel density estimation with a Gaussian kernel. The theoretical underpinnings of the descriptor are expressed in Eq. (3).

2 $$\begin{aligned} g_2(R_l, R_m)= & {} \frac{1}{\vert {R_l - R_m} \vert } \end{aligned}$$

3 $$\begin{aligned} {P_2}^{l, m}(x)= & {} \frac{1}{{\sigma _2 \sqrt{2\pi }}} e^{\frac{({x-g_2(R_l, R_m)})^2}{2\sigma _2^2} } \end{aligned}$$

where $R_l$ and $R_m$, refer to the Cartesian coordinates of atoms *l* and *m*, respectively, and $g_2$ is derived from the reciprocal of their Euclidean distances. As the distributions are calculated for a set of predefined values of *x* and standard deviation $\sigma _2$, each possible pair of the *k*-species present has multiple such values. These are combined into a singular value by taking the weighted average for each of these pairs, as expressed in Eq. (4).

4 $$\begin{aligned} {MBTR}_2^{Z_1, Z_2}(x) = \sum _{l}^{\vert Z_1\vert } \sum _{m}^{\vert Z_2 \vert } w_2^{l, m} \times {P_2}^{l, m}(x) \end{aligned}$$

where $Z_1$ and $Z_2$ are the atomic numbers for atoms *l* and *m*, respectively, and $w_2$ is the weighting function.

We use the DScribe implementation of the originally proposed method^58^. The exponential weighting function $(w_2 = e^{-sx})$ is used to keep the distributions tightly limited to atoms that reside in the neighbourhood. For that, a cut-off threshold of $1\times 10^{-2}$ and a scaling parameter of 0.75 are used^8^. A key parameter of the implementation, $n_{grid}$, refers to the number of discretization points and, in turn, determines the total number of features in the resulting vectors through Eq. (5). To determine its optimal value, we observe the correlation between the resulting vectors, $\text {MBTR}_{n_{\text {grid}}}$, for different $n_{\text {grid}}$ and the corresponding SASA values according to Eq. (6). These correlation scores are presented in Table 4.

5 $$\begin{aligned} n_{\text {features}} = \frac{n_{\text {elements}} \times (n_{\text {elements}} + 1)}{2} \times n_{\text {grid}} \end{aligned}$$

where $n_{\text {elements}}$ is the number of total elements encountered throughout the descriptor generation process; here, $n_{\text {elements}}$ = 8.

6 $$\begin{aligned} C_2 = \sum _{j=1}^{n} \left| \sum _{i=1}^{k} Corr({\text {MBTR}_{n_{\text {grid}}}}^{\langle i\rangle }, \text {SASA}) \right| \end{aligned}$$

where, *k* is the number of features and *n* is the number of samples used for the evaluation of $C_2$.

**Table 4:** Correlation to SASA for different values of $n_{\text {grid}}$.

$n_{\text {grid}}$ value	Number of features, $n_{\text {features}}$	Correlation score, $C_2$ (%)
2	72	**45.22**
3	108	42.17
4	144	43.85
5	180	44.39
6	216	43.73

The maximum value is in bold.

From Table 4, we can observe that the correlation scores do not vary much for different values of $n_{\text {grid}}$. However, as the lowest possible value of 2 for the parameter achieves the highest score while producing the smallest representation, it is chosen for this work.

### Time series model

For the time series model, we use two approaches: the first is based on a transformer model, while the second approach implements an ensemble of XGBoost models.

#### Transformer model

**Figure 6:**
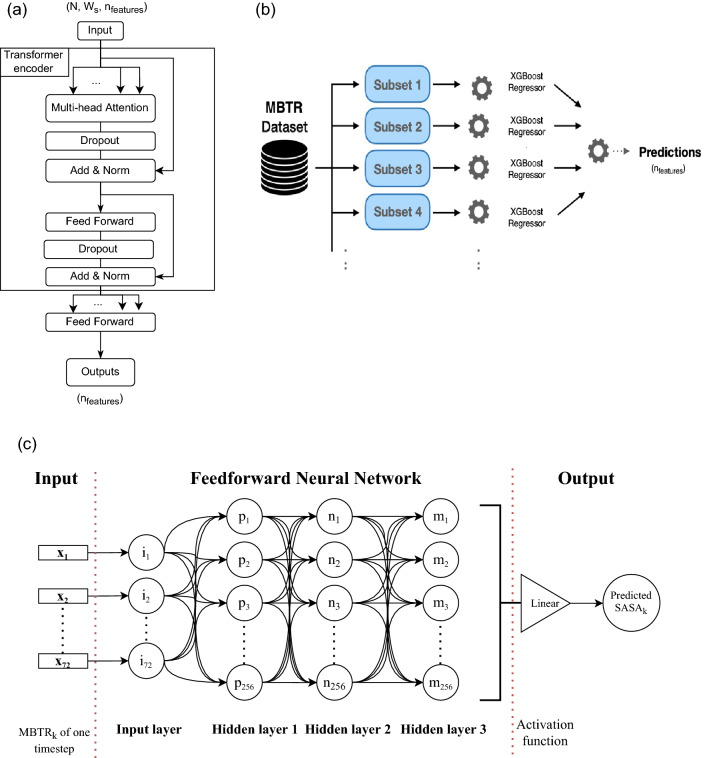
(**a**) Block diagram of the transformer model. Four different layers are used in the transformer model^59^. Multi-head attention allows the model to jointly attend to information from different representation subspaces at different positions. The dropout layer prevents overfitting, the normalization layer improves the training speed for various neural network models, and after normalization, the results are added to the input. The feedforward layer is a nonlinear mapping from an input pattern *x* to an output vector *y*. (**b**) Block diagram of the ensemble approach. The MBTR vector batches are split for each of the features, and all 72 subsets of data are used with an XGBoost regression model. The predictions from each model are then combined to produce the $n_{\text {features}}$-length output. (**c**) Block diagram of the SASA model. The 72 MBTR features at timestep *k* are passed to the *i* nodes of the input layer. The information in the input layer nodes is then passed to all the nodes of the hidden layers with *p*, *n* and *m* nodes interconnected in such a way that each node in the current layer is connected to every other node in the previous layer. The output is a single scalar value representing the SASA at timestep *k*.

A transformer is a model architecture whose structure combines an encoder and decoder. For this work, we use the encoder part of the model taking a batch of data with a fixed window size as input and outputting the multivariate vector of the MBTR corresponding to the next timestep. The architecture of the model is illustrated in Fig. 6a.

In this work, a multi-head attention mechanism is used with 12 heads, the size of each attention head is 256, and the dropout probability is 0.25. The normalization layer uses $\varepsilon = 1 \times 10^{-6}$ to normalize the input. The feedforward layer consists of a normalization layer, a 1-D convolutional layer, a dropout layer and another 1-D convolutional layer. The normalization layer and the dropout layer inside the feedforward layer use the same $1 \times 10^{-6}$ and 0.25 for the $\varepsilon$ and dropout probability, respectively. The first convolutional layer uses a ReLU activation layer with a kernel size of 1 and filters it into 4 outputs. The second convolutional layer also uses a kernel size of 1 and provides 1 output.

The model is trained by taking a window, $w_s$, and all the features, $n_{\text {features}}$, from each design in the training set and then combining them to predict the next $n_{\text {features}}$-length vector at the next timestep. For instance, providing the MBTR representing the first 40 timesteps of the MBTR as input will produce the MBTR for the 41st timestep by evaluating the learned pattern from the training dataset. This model takes 1378.5 s for training on a Tesla P100 PCIe 16 GB GPU with 28 2.4 GHz Intel Broadwell CPU cores and 230 GB of RAM.

#### Ensemble model

The second approach is described as an ensemble approach with an XGBoost regressor, by creating one model for each feature. The model works by training a window, $w_s$, of each feature to predict the next timestep’s value for the respective feature. The difference from the previous approach is that one feature of each design is taken to learn the pattern from it instead of taking the whole $n_{\text {features}}$ as input. As a result, it provides better predictability of the MBTR. Moreover, on the same hardware as the transformer model, the training time of this approach is 20.73 times faster. The architecture of this model is shown in Fig. 6b.

For instance, providing the MBTRs representing the first 40 timesteps as input, the first model of the ensemble approach only predicts the value for the first feature. The function then iterates through the other features, and for each feature, the corresponding model predicts the value for the next timestep. Finally, all predicted results are combined into one MBTR vector for the target timestep.

### SASA model

A limitation of using the MBTR is that the encoded data cannot be reverted to atomic coordinates. Therefore, it is not possible to calculate SASA values from the MBTR directly. However, as ML has the potential to identify and understand hidden relationships, we use a feedforward neural network to predict the continuous values of the SASA from the encoded data. The MBTR as the input data represents the state of the NP at one timestep. The training and testing datasets are divided in the same way as the time series model.

The proposed network consists of 4 dense layers: (i) an input layer with 256 neurons and ReLU as the activation function, accepts 72 MBTR features; (ii) 3 hidden layers, each with 256 neurons and ReLU as the activation function; and (iii) an output layer using a linear activation function on a single neuron suitable for the regression task. For training, the model iteratively passes over the whole training set 500 times, with a batch size of 32, and optimizes using the Adam algorithm at a learning rate of 0.0001. The resulting value represents the predicted SASA. The performance of this regression model is evaluated using the MAE error metric to evaluate how close the predictions are to the expected values in either direction. The architecture of the model is shown in Fig. 6c.

## Data Availability

The transformed data, MBTRs for all the NP designs used in this experiment are available at: https://github.com/Evonano-Team/evonano-ml/tree/master/data/processed. PDB files of the NP designs can be provided from the authors on reasonable request.

## Code availability

Code used in this project is available at: https://github.com/Evonano-Team/evonano-ml.
